# Comparative Analysis of the Full-Length Genome Sequence of a Clinical Isolate of *Human Parainfluenza Virus 4B*

**DOI:** 10.6064/2012/871201

**Published:** 2012-07-08

**Authors:** John A. Lednicky, Thomas B. Waltzek, Micah D. Halpern, Sara B. Hamilton

**Affiliations:** ^1^Department of Environmental and Global Health, College of Public Health and Health Professions, University of Florida, P.O. Box 100188, Gainesville, FL 32610-0188, USA; ^2^Emerging Pathogens Institute, University of Florida, Gainesville, FL 32610, USA; ^3^GenSol Diagnostics, 4945 Apollo Avenue, St. Cloud, FL 34773, USA; ^4^Medical Countermeasures Division, MRIGlobal, 425 Volker Boulevard, Kansas City, MO 64110, USA

## Abstract

We are engaged in airborne transmission and epidemiology studies of respiratory pathogens, with particular interest in *human parainfluenza virus* type 4 (hPIV-4) and other lesser studied viruses. In this paper, hPIV-4 was detected in primary rhesus monkey kidney (PRMK) cells that had been inoculated with nasopharyngeal swab material obtained from a child with a mild upper respiratory tract illness. Attempts to isolate the virus in pure culture were hampered by the presence of a fast-growing simian spumavirus that was a contaminant of the PRMK cells. Total RNA was extracted from the PRMK cell culture, and PCR followed by sequencing of a subgenomic section of the *fusion protein* gene suggested the hPIV-4 was subtype 4B. At the time of this work, two complete but dissimilar hPIV-4B genomes had been deposited by others in GenBank. To gain better insights on hPIV-4B, and to test methods that we are developing for viral forensics, the entire genomic sequence of our virus was determined from archived RNA. The hPIV-4B genomic sequence that we determined conforms to the paramyxovirus “rule of six.” Here, we compare and contrast the genetic features of the three completely sequenced hPIV-4B genomes currently present in GenBank.

 Human parainfluenza viruses (hPIVs) are single-stranded, negative sense RNA viruses of the genus *Rubulavirus*, family *Paramyxoviridae*, which cause acute respiratory tract infections in children and adults. Four hPIV serotypes (hPIV 1–4) have been identified; serotype 4 is further subdivided into two antigenic subtypes: 4A and 4B [1, 2]. The epidemiology and clinical manifestations of hPIV 1–3 are well known, whereas comparatively little is known about hPIV4s, as they are difficult to isolate in cell culture and are absent from routine respiratory virus detection tests in most clinical virology laboratories [3–5]. Whereas hPIV4s were formerly mostly associated with mild respiratory illnesses in young people, recent studies indicate the viruses can cause more severe infections such as pneumonia in young and older patients (mentioned in [3–5]).

The genomic cRNAs of hPIV-4 subtypes 4A and B are a little >17.0 kbp in length. Their viral genomes encode for nucleocapsid (NP), phospho (P), nonstructural (V), matrix (M), fusion (F), haemagglutinin-neuraminidase (HN), and large (L) proteins. Prior to this work, there were two complete hPIV-4B sequences in GenBank: those of strains 68–333 [6] and SKPIV-4 [7].

Primary monkey kidney (PMK) cells are inoculated with appropriate specimens for the detection of human parainfluenza viruses in many American diagnostic microbiology laboratories. The PMK cells available to these diagnostic laboratories are usually harvested from one of various *Chlorocebus* or Asian macaque species and contain a mixture of kidney cell-types. Furthermore, the PMK cells can contain endogenous simian viruses that are either latent in the kidneys or cause persistent but inapparent kidney infections in their hosts. Their presence in PMK cultures generally becomes evident after the cells are maintained in culture for more than a few days. Regardless, experience has shown that the probability of detecting human parainfluenza viruses in clinical specimens through *in vitro* virus culture is better with PMK cells other than cell lines commonly used in diagnostic virology laboratories. 

The virus analyzed in this work was from an immunocompetent two-year-old child in Chicago with a mild upper respiratory infection of two-days duration at the time of specimen collection (October 2004). At the time of specimen collection, the patient's symptoms included runny nose, barky cough, low fever, and decreased appetite. A nasopharyngeal swab specimen from the patient was eluted in universal virus transport medium (BD, NJ, USA), and equal aliquots of the solubilized material inoculated into A549, MDCK, WI38, and rhesus PMK cells and inoculated at 35°C. The PMK cell-culture media contained antibodies against PIV5 and SV40. The cultures tested negative by direct immunofluorescence assays (DFA) at 24 and 72 hrs p.i. using a commercial kit that detects PIV-1, -2, -3, influenza A and B viruses, adenovirus, and RSV (Respiratory Panel 1 DFA kit, Millipore, Billerica, MA, USA). However, with FITC-labeled anti-PIV-4 antibody (catalog item no. 5034, Millipore), sporadic PMK (but not the other) cells were borderline positive at 24 hr and positive at 72 hr p.i., demonstrating characteristic punctuate intracytoplasmic staining. Unfortunately, large vacuoles and widespread cell deterioration were evident in about 30% of the PMK cells by 72 hrs p.i. (including the negative controls), suggesting that a contaminating virus was present in the PMK cultures. Aliquots were therefore taken from the hPIV-4B-infected PMK culture and inoculated into NCI-292, Vero, LLC-MK2, or CV-1 cells, in hopes of isolating the hPIV-4 virus in cells not susceptible to the contaminating virus. Thereafter, an RNA stabilizing solution (RNAlater, Ambion, Austin, TX, USA) was added to PMK cells, total RNA purified as described previously [8], and the RNA archived at −80°C. Attempts to isolate hPIV-4 were not successful; the contaminant, identified as a Group VI spumavirus (foamy retrovirus) (data not shown), caused extensive CPE (large vacuoles) 24 hrs after inoculation of the NCI-292, Vero, LLC-MK2, or CV-1 cells, and all the cultures were terminated.

Two-step reverse transcription PCR of the archived RNA with primers Para4-F (5′-catgggtgtcaaaggtttatc-3′) and Para4-R (5′-tgctgctgtaacttgtgcagc-3′) amplified a 376-base pair (bp) section of the HPIV-4 *F* gene [8]. Sequencing of the amplicon revealed the virus was probably hPIV-4B. As a complete genomic sequence of hPIV-4B was not available for comparison in 2004, and our priorities were focused on other viruses, further analyses were postponed until an opportune time was available for the development of sequencing strategies appropriate for hPIV-4B. 

We revived our sequencing efforts after two independently-derived hPIV-4B sequences were deposited in GenBank. For our work, targeted hPIV-4B sequences were RT-PCR-amplified from the archived RNA using a genome walking approach. Overlapping primers described in [6, 7] and others purpose-designed by us for our tasks were used for PCR amplification and sequencing. Superscript II reverse transcriptase (Life Technologies) was used for first-strand cDNA synthesis in the presence of SUPERase-In RNase inhibitor (Ambion), and high fidelity Platinum *Taq *DNA polymerase (Life Technologies) was used for PCR. The 3′ and 5′ ends of the viral genome were determined from vRNA using a RACE (rapid amplification of cDNA ends) kit (RLM RACE, Ambion, Austin, TX) following the manufacturer's instructions. Of note, efforts for determining the 3′ and 5′ end sequences of the viral genome were laborious; these tasks are simpler using viral genomes arising from purified virus particles. Sequences were directly analyzed using an Applied Biosystem 3130 DNA analyzer by using BigDye Terminator (v. 3.1) chemistry and the same oligonucleotide primers used for amplifications. The virus sequence was designated hPIV-4B 04-13 (“04-13” signifies “unusual” isolate no. 13 of year 2004).

The complete hPIV-4B 04-13 cRNA is 17,304 bp and thus conforms to the paramyxovirus “rule of six” since it is divisible by 6. A full-genome BLAST analysis reveals 98% homology with hPIV-4B strain 68–333 and 97% homology with hPIV-4B strain SKPIV-4. Key genetic features of hPIV-4B strains SKPIV-4, 68–333, and 04-13 are given in Table 1. 

 The deduced amino acid sequences of the F, H-N, L, M, NP, and P from hPIV-4B isolate 04-13 were aligned against the homologous sequences from 7 other members of the genus *Rubulavirus* and 2 members of the genus *Avulavirus*. Sequence alignments were performed using Mafft 5.8 [9] followed by minor manual adjustments in ClustalW [10]. The E-INS-I alignment strategy was used with the following parameters: scoring matrix (BLOSUM62), gap open penalty (1.53), and offset value (0). For each gene alignment, the sequence was trimmed to the first conserved amino acid at the 5′ and 3′ ends prior to analyses. To assess gene concordance, Bayesian analyses were performed independently for each gene. Phylogenetic trees were constructed using MrBayes v. 3.1.2 [11]. A mixed prior was used on amino acid models and default priors for topology and branch lengths. The Markov chain was run for a maximum of 10 million generations, with a stopping rule implemented so that the analysis would halt when the average deviation of the split frequencies was <0.001%. Four independent analyses were conducted, each with 1 cold and 3 heated chains with the default heating parameter (temperature = 0.2). Every 50 generations were sampled and the first 25% of MCMC samples discarded as burn-in. 

 Preliminary phylogenetic analysis revealed that there was only a single significant incongruence among individual gene trees (defined by the presence of incompatible bipartitions that received a posterior probability of >90%, resp.). The incongruence involved the *M*-gene analysis that supported an alternate branching pattern for the mumps virus as has been previously observed [11]. Therefore, for the final analysis, we concatenated the sequences for the 6 genes into 1 matrix. The dataset contained 4442 amino acid characters (including gaps) for 10 viral taxa. The concatenated 6-gene Bayesian analysis demonstrated with a high level of confidence that hBIV4B isolate 04-13 is most closely related to hPIV-4B isolate 68–333 with hPIV-4B isolate SKPIV4 as the sister group to the other two isolates (Figure 1). *Human parainfluenza virus* 4A was found to be the sister group to the hPIV-4B isolates. The hPIV4 clade was found to be the sister group to a second *Rubulavirus* clade composed of mumps virus, simian virus 5, simian virus 41, and human parainfluenza virus 2.

 The results of our genomic level phylogenetic analysis are consistent with previous analyses of the genus *Rubulavirus* [6, 7, 12]. As pointed out by Yea et al. [7], the genome of SKPIV-4 does not follow the paramyxovirus “rule of six.” Theirs is not a sequencing error; paramyxovirus genomes that violate the rule are occasionally encountered ([7], and J. Lednicky, unpublished). It will be informative henceforth to determine if the presence of “aberrant length” hPIV-4B genomes (i.e., those genomes whose length is not divisible by 6) in virus isolated from sick individuals correlates with clinical presentation and also whether genomic alterations occur as a consequence of passage of hPIV-4B in the primate cells used for the detection of the viruses by diagnostic laboratories.

## Figures and Tables

**Figure 1:**
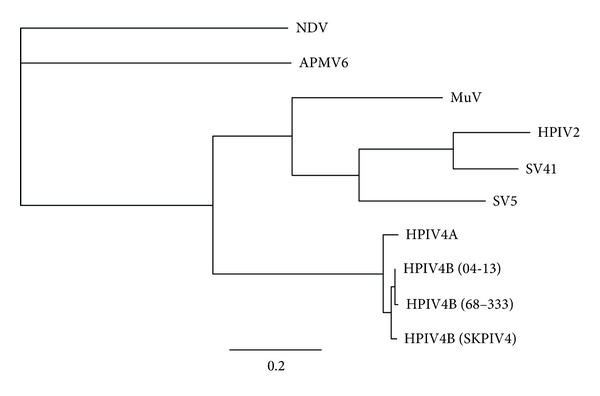
Phylogram depicting the relationship of hPIV-4B isolate 04-13 to representative members of the genus *Rubulavirus*. Tree based on the concatenated deduced amino acid sequences of the F, H-N, L, M, NP, and P proteins (4442 amino acid characters including gaps). Members of the genus *Avulavirus* (*Newcastle disease virus*; NDV and *Avian paramyxovirus 6*; APMV6) were included as outgroups. All nodes were supported by a posterior probability of 100. Branch lengths are based on the number of inferred substitutions, as indicated by the scale. Gene sequences were obtained from the complete genomic sequences for NDV (RefSeq accession no. NC_002617), APMV6 (NC_003043), *Mumps virus* (MuV; NC_002200), *human parainfluenza virus 2 *(HPIV2; NC_003443), *Simian virus* 41 (SV41; NC_006428), *Simian virus 5* (SV5; NC_006430), *human parainfluenza virus 4A* (HPIV4A; GenBank accession no. AB543336), hPIV-4B (04-13; AB543337, 68–333; JQ241176, SKPIV4; EU627591).

**Table 1:** Key genetic features of three fully sequenced hPIV-4B strains.

Virus designation		SKPIV-4	68–333	04-13
Genbank accession number		EU627591	AB543337	JQ241176.1
cRNA genome length		17,361 bp	17,304 bp	17,304 bp
Leader sequence^a^		>55 nt?	55 nt	55 nt
First 20 nt of viral genome complimentary to terminal 20 nt?		No	Yes	Yes
Putative 3′ peptide?		—	nt 52–75	nt 52–75
ORF 1 NP		551 aa	551 aa	551 aa
		nt 155–1,810	nt 101–1,756	nt 101–1,756
CTAAGAT in 1st intergenic region		Yes	Yes	Yes
ORF 2 P/V	P	399 aa	399 aa	399 aa
		nt 2,096–3,293	nt 2,041–3,238	nt 2,041–3,238
	V	229 aa	229 aa	229 aa
		nt 2,096–2,785	nt 2,041–2,730	nt 2,041–2,730
CAGAAGTA in 2nd intergenic region		Yes	Yes	Yes
ORF 3 M		382 aa	382 aa	382 aa
		nt 3,589–4,737	nt 3,531–4,670	nt 3,531–4,670
TCTGACACACAGCT AGAGCCA AATAC in 3rd intergenic region		TCTGACACACAACT-AAAGCCAAGCAT	Match	TCTGACACACAGCT-AGAGCCAAATAT
ORF 4 F		543 aa	543 aa	543 aa
		nt 5,232–6,863	nt 5,174–6,805	nt 5,174–6,805
CTATTAT follows poly A in 4th intergenic region		Yes	Yes	Yes
ORF 5 HN		579 aa	574 aa	574 aa
		nt 7,563–9,302	nt 7,506–9,230	nt 7,506–9,230
ORF 6 L		2,279 aa	2,279 aa	2,279 aa
		nt 10,025–16,864	nt 9,970–16,809	nt 9,970–16,809

^a^Leader sequence as defined by Komada et al. [6].
